# Prospective Roles of Extremophilic Fungi in Climate Change Mitigation Strategies

**DOI:** 10.3390/jof10060385

**Published:** 2024-05-27

**Authors:** Imran Ali, Hina Qaiser, Roheena Abdullah, Afshan Kaleem, Mehwish Iqtedar, Irfana Iqbal, Xiaoming Chen

**Affiliations:** 1 School of Life Science and Engineering, Southwest University of Science and Technology, Mianyang 621010, China; 2 Institute of Molecular Biology and Biotechnology, University of Lahore, Lahore 54000, Pakistan; 3 Institute of Biochemistry, University of Balochistan, Quetta 87300, Pakistan; 4 Department of Biology, Lahore Garrison University, Lahore 54000, Pakistan; hinaqaiser@lgu.edu.pk; 5 Department of Biotechnology, Lahore College for Women University, Lahore 54000, Pakistan; afshankaleem@yahoo.dk (A.K.); miqtedar@gmail.com (M.I.); irfana.ali@outlook.com (I.I.)

**Keywords:** bioremediation, carbon sequestration, climate change, extremophilic fungi, mitigation strategies

## Abstract

Climate change and the resultant environmental deterioration signify one of the most challenging problems facing humankind in the 21st century. The origins of climate change are multifaceted and rooted in anthropogenic activities, resulting in increasing greenhouse gases in the environment and leading to global warming and weather drifts. Extremophilic fungi, characterized by their exceptional properties to survive extreme habitats, harbor great potential in mitigating climate change effects. This review provides insight into the potential applications of extremophilic fungi in climate change mitigation strategies. They are able to metabolize organic biomass and degrade carbon compounds, thereby safely sequestering carbon and extenuating its release into the environment as noxious greenhouse gases. Furthermore, they possess extremozymes, which break down recalcitrant organic species, including lignocellulosic biomass and hydrocarbons. Enzymatic machinery equips these extremophilic fungi to perform the bioremediation of polluted environments. Extremophilic fungi can also be exploited for various biological interventions, such as biofuels, bioplastics, and other bioprocessing applications. However, these fungi characterize a valued but underexplored resource in the arsenal of climate change mitigation strategies.

## 1. Introduction

**Figure 1:**
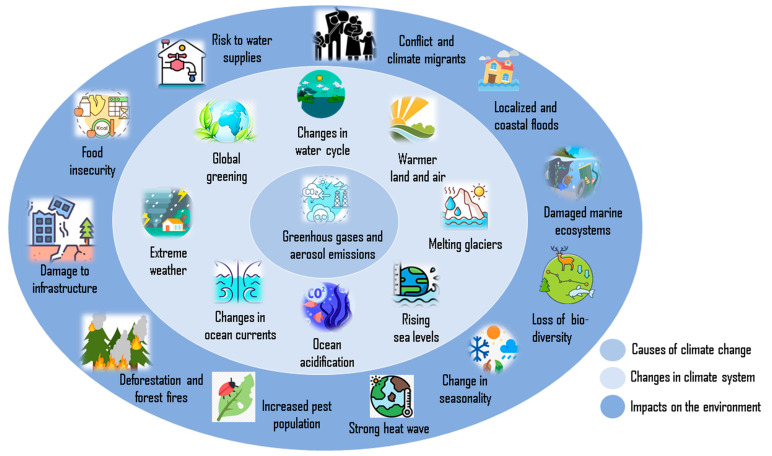
Summarized depiction of climate change causes and their impact on the environment.

The scientific, political, and economic communities of the world face a huge task in combating climate change. Anthropogenic greenhouse gas emissions have become excessive due to the industrial era’s mass use of fossil fuels and the exponential growth of the world’s population. Together with other human-caused variables, their actions exacerbate climate change, the greatest threat to natural ecosystems and the goods and services they provide (Figure 1). Because of its effects on different parts of the world’s landmass, climate change has recently emerged as a major global issue. Heat waves are becoming more frequent and more intense, cold temperature extremes are becoming less common, and precipitation patterns are changing as a result of these impacts. The global mean temperature might rise by 1.5 °C by the end of the twenty-first century due to increased atmospheric greenhouse gas levels. [1]. Carbon dioxide (CO_2_) is the most important greenhouse gas, as it accounts for 76% of all greenhouse gases. The atmospheric concentration of carbon dioxide has risen from 280 parts per million at the beginning of the industrial revolution [2] to 410 parts per million as of March 2019.

Deforestation, forest fires, fossil fuel consumption, vehicle use, and many other human-caused activities are rapidly increasing the rate of CO_2_ buildup. Natural carbon dioxide sinks mostly consist of water, plants, and soil. Recent years have seen significant progress in the scientific community’s attempts to reduce atmospheric CO_2_ concentrations and so lessen the impact of climate change and global warming via carbon sequestration [3]. At high latitudes, temperature swings are the most pronounced; specifically, predicted shifts in the amount of snow and sea ice in the Arctic Ocean disrupt the surface energy balance in terms of albedo (reflectivity) in a significant way. Hence, the tropics, where substantial changes to the surface’s basic characteristics are not expected to happen to the same degree, would not experience the strong positive feedback on the climate system that high-latitude areas would. In tropical deforestation areas, however, this is not always the case since the albedo, thermal, and humidity properties of the earth are changed when rainforests are replaced with controlled crops and trees. This changes the climate on a regional and continental scale.

The far higher thermal capacity of water causes the amplitude of temperature changes over continents to be substantially larger than that overseas. According to Rastogi et al., (2002), soil respiration is responsible for 20% of the total atmospheric carbon dioxide emissions, whereas soil sequestration is responsible for storing carbon [2]. Global soil carbon sequestration is 3.3 times more than atmospheric carbon sequestration (2500 Gt C) [4]. Out of a total of 2500 Gt, 1550 Gt is retained by soil organic matter (SOC), and 950 Gt is retained by soil inorganic carbon. Biomass contains 4.5 times less carbon (C) than soil on the earth [5]. Soil carbon sequestration relies heavily on the biological soil crust. In many ecosystems, soil fungi, particularly arbuscular mycorrhizal fungi, increase soil carbon sequestration. Our ability to raise enough food to meet the needs of our fast-expanding population will be severely limited by the effects of climate change. Due to the increasing prevalence of drought and salinization in Mediterranean-type ecosystems (MTEs), there is an immediate need to enhance wheat productivity in light of these changing circumstances [6]. The presence of fungal endophytes in extreme conditions has the potential to increase agricultural yields, resistance to pests and diseases, and overall chances of survival.

### Extremophiles at the Interface of Climate Change

**Figure 2:**
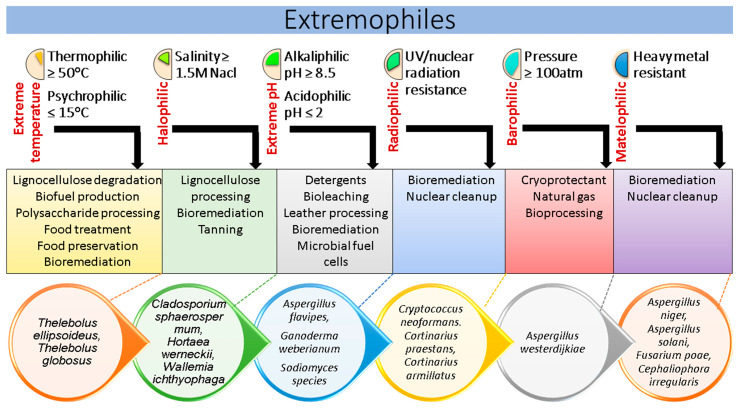
Summarized extremophiles and their diverse roles in sustainable environments.

Extremophiles offer exceptional ecosystem services (Figure 2) and are a rich source of enzymes, which demonstrate enhanced thermostability and adaptability to changes in pH, water activity, and temperature. These enzymes exhibit remarkable structural flexibility and are particularly effective under extreme conditions. Additionally, extremophiles secrete stress-tolerant proteins, which derive their name from the organisms that require them, which may find industrial applications or be economically significant. An excellent source of novel drugs with antimicrobial, antiviral, and anticancer activity is one of the primary reasons why they are of such interest to scientists [7]. Though research on extremophiles and climate change is in its early phases, new adaptations within microbial communities are certain. The melting of permafrost, deserts, sea ice, and boreal forests is causing the extinction of many species of fungi that have not yet been named. It is of utmost importance to discover extremophiles and study their possible uses in biotechnology, agriculture, and the reduction in abiotic stress since desertification and the rise in water-stressed environments are both accelerated by climate change.

## 2. Biological Variability in Fungi and Its Significance

**Figure 3:**
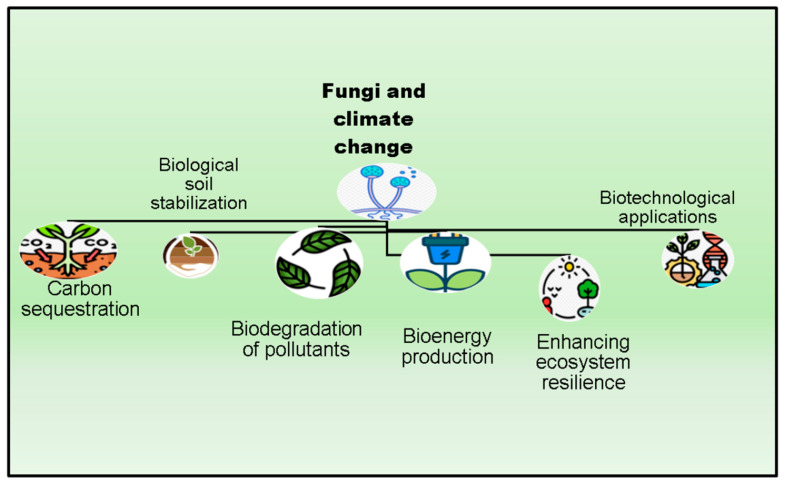
Role of fungi in mitigating climate change effects.

Fungi recycle the majority of organic resources for the advantage of other organisms. Some fungi are more discriminating than others when it comes to the organic compounds they can degrade. Recent research has focused on two main areas: the impact of climate change on fungi and the role that fungi may play in reducing that impact [8]. The rising atmospheric concentrations of greenhouse gases are the primary driver of climate change, an environmental catastrophe on a worldwide scale. This phenomenon has several manifestations, such as warmer average temperatures and more intense and frequent weather events. There may be regional variations in the effects of climate change, as expected. Hot springs, hydrothermal vents, and other environments with very high or low pH are home to fungi that, if used properly, might mitigate the impacts of climate change in ecosystems other than those on land and in the Arctic. Extreme environments are fascinating to examine because of the distinct variety of microbes that they support. Changes in moisture and temperature are two aspects of climate change that are likely to have an effect on fungi, causing distribution shifts and ecological disruption [1]. Fungi are also capable of contributing to the mitigation of climate change’s negative impacts (Figure 3). As an illustration, specific fungal species possess the ability to decompose and retain organic compounds, thereby potentially functioning as a carbon sequestration and storage mechanism [9].

The decomposition of organic matter in extreme environments, including hot springs, acidic soils, and deep-sea vents, is the domain of extremophilic fungi. They contribute to the global carbon cycle by emitting carbon dioxide (CO_2_) into the atmosphere during the process of organic material decomposition. Climate change can have an influence on carbon cycling processes by causing alterations in the distribution and activity of extremophilic fungi.

Some extremophilic fungi are capable of influencing climate change-related feedback cycles. For example, the decomposition processes of extremophilic fungi may experience heightened activity in regions with warming permafrost. The problem of global warming may be worsened as a consequence of the increased emissions of greenhouse gases, such as CO_2_. The possible reactions and adaptations of creatures to environmental changes caused by climate change may be better understood by studying the processes by which extremophilic fungi adapt to harsh environments. One possible strategy to lessen the impact of climate change on other ecosystems and creatures is to study extremophilic fungi that are resistant to harsh environments (Table 1). Researchers are also looking at the possibility of using fungi to make bioplastics, biofuels, and other bio-based products that help create a greener, lower carbon economy [10,11].

There exists an intrinsic connection between forests and climate change due to the manner in which forest trees sequester carbon in the form of tree biomass and remove carbon dioxide (CO_2_) from the atmosphere via photosynthesis. Consequently, forests play a substantial role in the mitigation of climate change. Soil serves as a significant sink for carbon, retaining an estimated two-thirds against turnover through the formation of chemical complexes or soil aggregates. Fungi, no doubt, also hold great potential in achieving the United Nations sustainable development goals, which protect the planet and make it a better place to live in peace and prosperity.

### 2.1. Carbon Sequestration by Fungi

**Figure 4:**
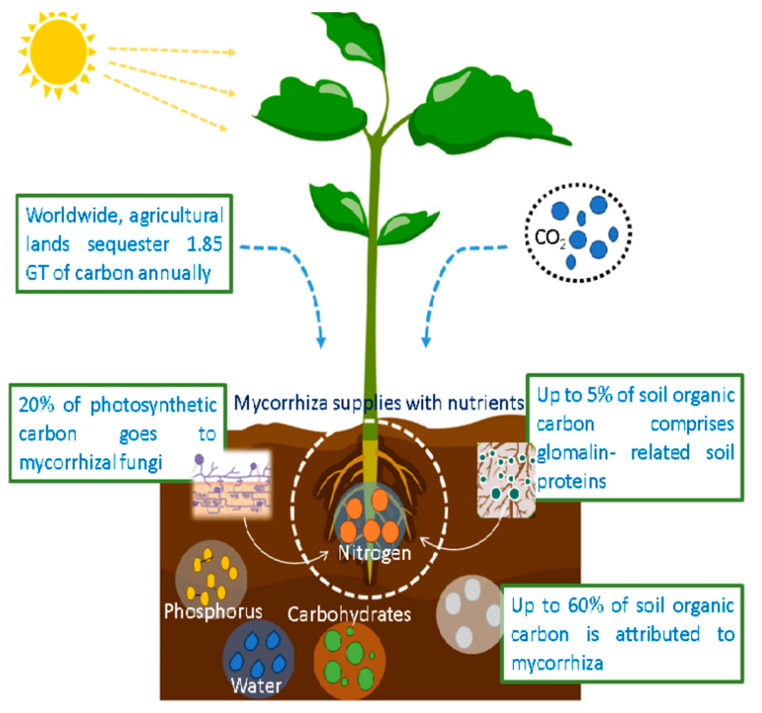
Carbon sequestration by mycorrhizal fungi.

Carbon serves as the fundamental constituent in every biological organism. Composed of CO_2_ in the atmosphere and dissolved in water, it is present in numerous forms, including soil organic matter, plant detritus, and gas. The carbon content of soil exceeds that of both the atmosphere as a whole and plant biomass. An abundance of diminutive fungi inhabits the soil, comprising hyphae that construct themselves from carbon. Upon this demise, this hypha decomposes rapidly, retaining its carbon content as soil organic matter for an extended period of time [38]. Carbon sequestration is the permanent retention of carbon in terrestrial biomass, the ocean, or the soil in order to impede or diminish the accumulation of carbon dioxide in the atmosphere. According to the Food and Agriculture Organization (FAO), carbon sequestration can be defined as the secure storage and capture of carbon that would have been released into the atmosphere or retained therein (FAO 2000). Fungal carbon sequestration is a substantial contributor to the mitigation of global warming in the northern hemisphere. Fungi establish symbiotic relationships (mycorrhizal fungi) with plant roots in the soil, thereby facilitating nutrient uptake by the plants. Mycorrhizal fungi thus promote plant growth, which accelerates the process of atmospheric carbon dioxide elimination via conversion to plant biomass (Figure 4). Alternative methods exist for the conversion of atmospheric carbon dioxide into plant biomass [39]. Soil carbon is enriched with organic matter from both above- and below-ground sources, such as roots, flowers, stems, leaves, and other plant debris. Furthermore, exudates are a kind of organic compounded matter that plants release into the soil. This process is known as rhizodeposition. Another major source of carbon for higher plants is rhizodeposition, which varies with the root architecture, surroundings, physiology, biochemistry, and chemical makeup of the organic matter that is deposited. Depending on the ecosystem’s climax community, the amount of waste input varies in size. The physicochemical features of the soil and bacteria determine how the underground input mostly adds to the organic matter and how long it stays stable [40,41,42].

The only factors that determine carbon sequestration are the autotrophs’ photosynthetic rate and the respiratory losses of both the autotrophs and their symbionts, which are mycorrhizal fungi. Based on their estimates, these fungi account for 5–20% of the symbiotic system’s net primary production [43]. When changes occur in both the absorption and degradation rates of carbon at the same pace and magnitude, an organization’s carbon sequestration capacity may be entirely reversed. The breakdown of carbon that is deposited in the root zone by microbes is a key process. These bacteria either live in or are connected to certain types of plants and soil. The degree of rhizoassociation also makes a big difference. Mycorrhizal fungi and other rhizoassociates do not contribute much to respiratory losses on their own. As a result, the exact calculation of mycorrhizal fungi’s carbon sequential potential has not been finalized yet. Some controlled and field studies have shown that photosynthesis and respiration in plants are necessary for mycorrhizal fungi to sequester carbon [43,44,45]. Below-ground respiration releases around 24 Pg C y^−1^ from soils in forests throughout the world into the atmosphere, with mycorrhizal fungi being responsible for about 16% of this amount [46,47]. Mycorrhizal fungi may increase their sequestration capability, which means they can drastically decrease the contribution [7]. Because of their remarkable adaptability, fungi can survive in environments that are not ideal for them. Fungi can adapt to a wide range of pH and temperature levels, making them ubiquitous in the natural world [48]. The carbon sequestration activity of fungi may be influenced by both biotic (involving other organisms) and abiotic (physical disturbance, soil temperature, pH, texture, moisture, and salt) variables.

In certain habitats and places, fungi may acquire organic matter and break it down in the soil, two of its main roles, which might greatly increase atmospheric carbon dioxide sequestration. Regardless, how exactly sequestration works is still a mystery. The quantifiable contribution of soil and mycorrhizal fungi to different ecosystems and relationships has not been evaluated or published yet. Soil and mycorrhizal fungal carbon sequestration trajectories may be better understood with this measurement. Moreover, the carbon sequestration potential can be increased by reducing the respiratory losses of mycorrhizal fungi and soil. Consequently, this area warrants further investigation in the future. Various soil types and mycorrhizal fungi, in addition to the function and potential of diverse fungal communities, may contribute to the enhancement of carbon sequestration in distinct ecosystems. Soil moisture, soil temperature, CO_2_ enrichment, rhizodeposition quality, and precipitation variations are among the elements that influence fungal growth and, by extension, the control of heterotrophic respiration. Soil and mycorrhizal fungi have evolved to adapt to certain environments, and we can learn a lot about those adaptations by studying how these factors affect them. When it comes to soil fungal respiration, there is a severe lack of research and understanding of the relationships between soil temperature, moisture, mycorrhizal diversity, and mycorrhizal composition and diversity. The extent to which mycorrhizal association contributes to carbon storage in soil that is contaminated, mineral-rich, or nutrient-rich is unknown.

Consequently, further investigation in this field may contribute to a more comprehensive comprehension of the mechanisms underlying fungal carbon sequestration.

### 2.2. Biological Soil Stabilization by Fungi

Biological soil stabilization by fungi pertains to the mechanism through which these fungi aid in the development and sustenance of stable soil structures, even in the most severe or hostile conditions. This phenomenon holds significant importance in areas that are susceptible to soil erosion, including deserts, arid regions, and degraded landscapes. Fungi typically construct elaborate networks of hyphae, which are filamentous structures constituting the fungal body. By penetrating the soil matrix, these hyphal networks bond to soil particles and produce a cohesive soil structure. This network stabilizes the soil by acting as a tangible barrier against wind and water erosion [49]. Certain species of extremophilic fungi generate extracellular components that aid in the adhesion of soil particles, including polysaccharides, glycoproteins, and glomalin-like compounds [50]. These substances serve as adhesives, promoting soil stability by cementing soil aggregates. Extremophilic fungi’s ability to generate extracellular matrices is crucial in environments depleted of organic matter or characterized by soil degradation. Fungi protect plants from deleterious microorganisms that may affect the soil’s health [51]. Arbuscular mycorrhizal (AM) fungi enable plants to withstand adverse physical and biological conditions [52]. Various investigations suggest the positive potential of AM fungi to help plants survive harsh environments such as deserts. [53,54]. As per the extensive hyphal networks, AM fungi can reach through the soil particles and enhance the water uptake ability of plants from below the ground [55]. These fungi can also influence the moisture-retaining characteristic of soil by enhancing the soil aggregates [56,57]. The bioaugmentation of AM fungi with *Azospirillum brasilense* boosts soil stabilization in the desert and prevents soil erosion [58,59].

### 2.3. Extremophilic Fungal Endophytes for Improved Crop Performance

In order to forecast the ramifications of global climate change on agricultural systems across the globe, it is vital to comprehend the degree of resilience that crops exhibit towards environmental duress. However, existing research on crop resilience has predominantly concentrated on the physiological and molecular reactions of plants. Understanding how crop physiology responds to changes in water availability [60], temperature [61], salinity [62], atmospheric CO_2_ concentrations, and soil chemistry has been the subject of considerable research in agricultural systems [63].

Persisting human population growth is presently causing an increase in food demand [64]. Projections indicate that by 2050, the global population will have grown to 9.7 billion [65]. In order to satisfy the escalating demand, it is imperative that the worldwide agricultural output increases by a minimum of 50% relative to its present level by that time [66]. However, by diminishing crop survival, quality, and productivity, climate change is likely to exacerbate food security issues [67]. Particularly threatened is crop productivity in Mediterranean-type ecosystems (MTEs), which supply a substantial portion of the world’s food and experience hot, dry summers [68]. Air temperatures have risen, and precipitation has decreased in MTEs as a result of climate change over the past century [69]. Climate models predict that by the end of the twenty-first century, MTEs will experience additional warming and decreased precipitation [70]. Significantly affecting MTEs, high evapotranspiration in the summer and spring, combined with warming and drought, drastically reduce water availability and cause soil salinization [71]. Consequently, in order to satisfy forthcoming food requirements, it is critical to enhance the productivity and adaptability of cereals cultivated in MTEs to climate change [72].

An example of the application of beneficial microorganisms to enhance agricultural productivity is fungal endophytes [73]. Endophytes exhibit a wide range of advantages that differ depending on the indigenous terrestrial environment [74]. They colonize nearly all plants in natural terrestrial ecosystems. Soil endophytes originating from arid and saline environments, including desert, montane, and Antarctic regions, could potentially improve agricultural productivity in MTEs [75]. Because of their inhospitable conditions and geographic isolation, these uncharted environments remain largely unexplored. However, they are valuable reservoirs of endophytes [76], which have the potential to produce an abundance of natural products that can improve the stress tolerance of hosts [77]. Verma et al., (2021) suggested that plants cultivated in extreme environments might contain novel endophytes that have the potential to impart environmentally favorable and sustainable benefits to crops [78]. The endophytes that have been investigated thus far have steadily elevated the levels of proline and phenols in the foliage of cereals that have been subjected to drought and salinity. Phenols serve as signaling molecules within the symbiotic relationships between plants and microbes [79]. On the other hand, proline, an amino acid associated with stress and produced by numerous fungi, including *Penicillium species* [80], is known to participate in antioxidant mechanisms and enhance drought tolerance. As a compatible osmolyte, the amino acid modulates the stability of macromolecules, proteins, and antioxidant enzymes and the equilibrium of intracellular redox homeostasis; it also serves crucial stress-protective functions [81]. Enhanced membrane stability, which proline likely induces, may, therefore, partially account for the increased survival and yield of crops subjected to salinization and drought, as well as the decreased lipid peroxidation in the foliage of inoculated plants exposed to the climate change scenario.

Research has identified the endophytic growth of *Penicillium* species in the tissues of a minimum of 117 plant species across 63 families. This includes numerous commodities, including coffee, quinoa, rapeseed, cotton, and common grapevine [82]. The proliferating presence of genus members within plant tissues implies that they might have the potential to enhance the stress tolerance of various economically significant commodities. The rapid generation and comparatively low cost of the sufficient biomass of these fungi for inocula are possible due to the simplicity of cultivating them on artificial media. Therefore, they are likely more appropriate for incorporation into bioformulations compared to certain other microorganisms, particularly arbuscular mycorrhizal fungi (AMF). By influencing water relations, phosphorus acquisition, and pathogen resistance, these fungi, which are members of the *Glomeromycotina* group, improve crop performance in moderately arid soils [83]. Nevertheless, the lack of plant hosts for AMF growth poses a substantial barrier to producing adequate quantities of inoculant material for crops, which is essential for increasing yield [84]. In addition, their presence in roots is scarce in extreme environments, such as desert, Antarctic, and montane soils, where aridity and/or salinity inhibit their formation [85,86]. On the other hand, ascomycetes, belonging to the *Aspergillaceae* family, including *Penicillium* species, are prevalent in arid and chilly environments [87]. This finding lends credence to the notion that hostile surroundings could potentially offer an abundance of advantageous endophytes [76]. To further investigate the potential of extremophile fungi to improve worldwide food security through sustained or increased food production [77], additional research is required to comprehend the intricate relationship between microbial inoculants, crop plants, the soil and plant microbiome, and the subsequent eco-physiological and biochemical responses of plants.

The idea that crops may withstand more biotic and abiotic challenges thanks to plant–microbial mutualisms, especially those involving arbuscular mycorrhizal fungi, is widely held [88]. Fifty percent of the studies that have looked into fungal root colonization due to drought have found it; nevertheless, researchers have seen more cases of increased colonization than decreased colonization. Because certain fungal species may have evolved to withstand water stress, specifically in the monoculture system, the overall structure of the fungal and AMF communities may be unaffected by temporary water scarcity. According to Li et al., (2019), arbuscular mycorrhizal fungi may reduce dehydration stress in C3 plants by improving their stomatal conductance compared to C4 plants, making them a potentially important deterministic species in climate change [89]. It is clear that a new species of *Wallemia peruviensis* is associated with agricultural areas that have a water activity of 0.84 and include kasmotrophic chemicals [90]. The investigation into the creation of tailored remedies for drought control is an essential area of study. For example, according to Le Pioufle and Declerck (2018), 70% of the extra radical hyphae kept up their metabolic activity even when there was a water shortage in in vivo trials on *Medicago truncatula* involving *Rhizophegus irregulars* MUCL 41,833 [91]. This recommends the need to look into other drought-resistant genotypes. Boerstler et al., (2010) found that there were more *Glomus intraradices* haplotypes in tilled agricultural areas compared to species-rich seminatural grasslands [92]. Additionally, *Glomus* sp. may change their sporulation or colonization processes to survive bad circumstances, according to Picone (2000), who also said that they are adaptable in their response to environmental changes [93].

### 2.4. Biological Interventions Based on Fungi for Sustainable Environments

**Figure 5:**
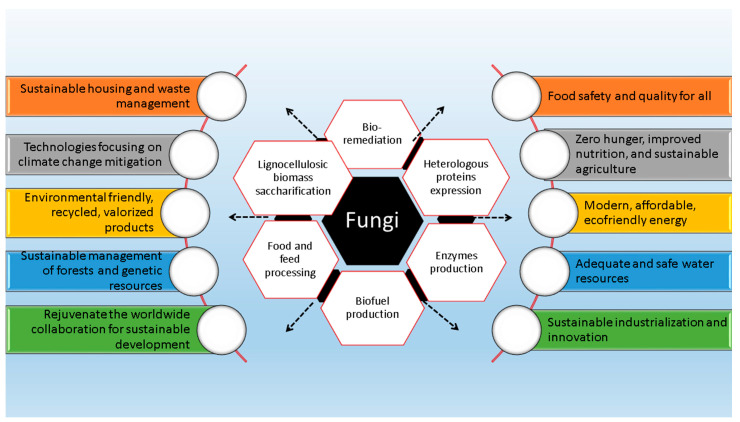
Biotechnological applications of fungi and their contribution to United Nations’ sustainable development goals.

In their capacity to decompose organic matter, fungi have a crucial skill that allows them to build ecologically responsible technology (Figure 5). Wood, paper, and textiles are just a few of the many organic things that may be biodegraded by the many fungi that inhabit the earth. As this process is fundamental to ecosystems’ natural nutrient recycling, it may be integrated into waste management and recycling systems to reduce trash’s environmental effects. For almost a hundred years, fungi have been used in biotechnology. Textiles, leather, and even certain polymers might be replaced by materials made from mycelia. Substituting materials obtained from mycelia for those derived from petroleum would significantly reduce plastic pollution. In the laboratory, scientists have also found and used fungi that break down plastics. In addition, studies have shown that basidiomycete enzymes may be used to break down plastic [94,95,96]. In recent years, researchers from many fields have been working together to develop composite materials from agricultural and forestry waste [97,98].

The fuel, food, feed, detergent, pulp and paper, chemical, and pharmaceutical sectors rely on enzymes and metabolites produced by fungi as they decompose plant waste. A vast array of goods may be produced by fungi, including proteins, vitamins, organic acids, chemicals, and even vegan leather [98]. Specifically, ligninases have been produced from the fungi *Pleurotus ostreatus* and *Phanerodontia chrysosporium*; these enzymes break down lignin and other resistant organic compounds while also cleaning polluted water and soil [99,100,101]. The use of fungi in waste management is not the only renewable energy source that has been studied. Biofuels like ethanol can be made from lipid-producing oleaginous fungi like *Aspergillus fumigatus, Umbelopsis isabelline, Thermothelomyces thermophilus,* and *Thermothielavioides terrestris* [102,103]. As a technical boon, biodiesel production has the potential to provide an eco-friendly alternative to conventional fossil fuels. Biogas, a combination of carbon dioxide and methane, may also be produced by the anaerobic digestion of lignocellulosic waste by fungi. In many parts of the globe, biogas is a major renewable energy source, and its production may significantly cut the emissions of greenhouse gases [104].

## 3. Conclusions

Although the amount of carbon found in soil is more than that found in the atmosphere, it is important not to ignore the possible influence that rising temperatures brought on by global warming might have on the variety and activity of fungi. There are a number of variables that determine the rate at which fungi sequester carbon from the soil, including the quality and quantity of hyphae in the soil, the texture of the soil, the amount of moisture in the soil, the management of the soil, the amount of physical disturbance, the use of pesticides, and other indirect and direct ecosystem factors. Important strategies for addressing climate change include the removal of carbon dioxide from the atmosphere or emission point sources via biotic or abiotic processes (photosynthesis), the creation of fuels with a carbon content that is either very low or nonexistent, and the limitation of the use of fossil fuels. A sustainable method for reducing the amount of carbon dioxide in the atmosphere is the sequestration of carbon in the soil by organisms.

## Figures and Tables

**Table 1:** Examples of fungal endophytes having plant growth-promoting effects under various stress conditions.

Fungal Species	Source Plant	Targeted Plant	References
Heat-Stress Tolerant			
*Aspergillus glaucus*	*Euphorbia indica* L.	*Glycine max, Helianthus* sp.	[12]
*Chaetomium* sp.	*Lasiurus scindicus*	*Oryza sativa*	[13]
*Curvularia crepinii*	*Hedyotis diffusa*, *Trifolium repens*, *Digitaria ischaemum*, *Silene tenuis*, *Cynodon dactylon Alternanthera philoxeroides*	*Oryza sativa*	[14]
*C. protuberata*	*Dichanthelium lanuginosum*	*Solanum* *lycopersicum*	[15]
*C. protuberata*	*Dichanthelium lanuginosum*	*Leymus mollis*, *Dichanthelium lanuginosum*, *Solanum lycopersicum*, *Oryza sativa*	[16]
Salt-Stress Tolerant			
*Aspergillus terreus*	*Chenopodium album* L.	*Triticum aestivum*	[17]
*Cochliobolus lunatus*	*Mirabilis jalapa* L.	*Ablemoschus esculentus* L.	[18]
*Cunninghamella bertholletiae*	*Solanum lycopersicum*	*Solanum lycopersicum*	[19]
*C. brachyspora SCb*	*Oryza sativa* L.	*Oryza sativa* L.	[20]
*Fusarium* sp. *V-4J*	*Pokkali Rice* varieties IR-64 and JBT 36/14.	*Oryza sativa* (Pokkali)	[21]
*Fusarium oxysporum*	*pomea pescaprae*	*Oryza sativa* L. variety-IR-64	[22]
*Glomus mosseae, G. intraradices, G. etunicatum*	*Acacia gerrardii*	*Cucumis sativus* L.	[23]
*Penicillium chrysogenum*	*Axonopus purpusii*	*Zea mays*	[24]
*Penicillium olsonii A3*	*Aleuropus littoralis*	*Nicotiana tabacum*	[25]
*Penicillium* sp. *NAUSF2*	Coastal rhizospheric soil (South Gujarat, India)	*Vigna radiata* L. cv. Co-4	[26]
*Periconia mascrospinosa, Neocamarosporium goegapense, N. chichastianum*	*Seidlitzia rosmarinus (Boiss.)*, *Zygophyllum eichwaldii (C. A. Mey.)*, *Haloxylon ammodendron (C. A. Mey.)*	*Cucumis sativus* L.	[27]
*Thermomyces longibrachiatum*	Saline-alkali soil	*Vigna unguiculata*	[28]
*Trichoderma longibrachiatum T6*	*Triticum aestivum* roots	*Triticum aestivum*	[29]
Drought Tolerant			
*Alternaria alternata*	*Elymus dahuricus*	*Triticum aestivum* L.	[30]
*Cunninghamella bertholletiae*	*Solanum lycopersicum*	*Solanum lycopersicum*	[19]
*Curvularia brachyspora SCb*	*Oryza sativa* L.	*Oryza sativa* L.	[20]
*C. protuberata*	*Dichantelium lanuginosum*	*Oryza sativa*	[31]
*Paecilomyces formosus LHL10, Penicillium funiculosum LHL06*	*Cucumus sativus* L. and *Glycine max* roots	*Glycine max*	[32]
*Periconia mascrospinosa, Neocamarosporium goegapense, N. chichastianum*	*Seidlitzia rosmarinus (Boiss.)*, *Zygophyllum eichwaldii (C. A. Mey.)*, *Haloxylon ammodendron (C. A. Mey.)*	*Cucumis sativus* L.	[27]
*Piriformospora indica*	*Prosopis juliflora* (Swartz) DC. and *Zizyphus nummularia* (Burm. fil.)	*Arabidopsis thaliana*	[33]
*Porostereum spadiceum AGH786*	*Glycine max* roots cv. Hwangkeumkong	*Solanum lycopersicum*	[34]
*Thermomyces harzianum ThSM3a (three strain consortium)*	*Annual grasses* (arid región CA, USA)	Corn and cotton	[20]
*Thermomyces harzianum 81Y1 and Fusarium solani 19 K3*	Roots of desert trees (Iran)	*Zea mays* L. cv. Simon	[35]
Biocontrol Agent			
*Aureobasidium subglaciale*	Subglacial ice	Golden delicious apples *(Malus domestica)*	[36]
*Aureobasidium pullulans, Aureobasidium melanogenum, Aureobasidium subglaciale*	Culture collection	Tomatoes and grapes	[37]

## References

[1] Malyan S.K., Kumar A., Baram S., Kumar J., Singh S., Kumar S.S., Yadav A.N., Yadav A.N., Singh S., Mishra S., Gupta A. (2019). Role of Fungi in Climate Change Abatement through Carbon Sequestration.

[2] Rastogi M., Singh S., Pathak H. (2002). Emission of carbon dioxide from soil. Curr. Sci..

[3] Bhattacharyya R., Bhatia A., Das T., Lata S., Kumar A., Tomer R., Singh G., Kumar S., Biswas A. (2018). Aggregate-associated N and global warming potential of conservation agriculture-based cropping of maize-wheat system in the north-western Indo-Gangetic Plains. Soil Tillage Res..

[4] Kumar S., Malyan S. (2016). Nitrification inhibitors: A perspective tool to mitigate greenhouse gas emission from rice soils. Curr. World Environ..

[5] Lal R. (2004). Soil carbon sequestration impacts on global climate change and food security. Science.

[6] Ballesteros G.I., Newsham K.K., Acuña-Rodríguez I.S., Atala C., Torres-Díaz C., Molina-Montenegro M.A. (2024). Extreme environments as sources of fungal endophytes mitigating climate change impacts on crops in Mediterranean-type ecosystems. Plants People Planet.

[7] Ali F.S., Akbar A., Prasongsuk S., Permpornsakul P., Yanwisetpakdee B., Lotrakul P., Punnapayak H., Asrar M., Ali I. (2018). Penicillium imranianum, a new species from the man-made solar saltern of Phetchaburi province, Thailand. Pak. J. Bot..

[8] Hyde K.D., Xu J., Rapior S., Jeewon R., Lumyong S., Niego A.G.T., Abeywickrama P.D., Aluthmuhandiram J.V., Brahamanage R.S., Brooks S. (2019). The amazing potential of fungi: 50 ways we can exploit fungi industrially. Fungal Divers..

[9] Jörgensen K., Granath G., Strengbom J., Lindahl B.D. (2022). Links between boreal forest management, soil fungal communities and below-ground carbon sequestration. Funct. Ecol..

[10] Oberti I., Paciello A. (2022). Bioplastic as a Substitute for Plastic in Construction Industry. Encyclopedia.

[11] Saye L.M., Navaratna T.A., Chong J.P., O’Malley M.A., Theodorou M.K., Reilly M. (2021). The anaerobic fungi: Challenges and opportunities for industrial lignocellulosic biofuel production. Microorganisms.

[12] Ali A.H., Abdelrahman M., Radwan U., El-Zayat S., El-Sayed M.A. (2018). Effect of Thermomyces fungal endophyte isolated from extreme hot desert-adapted plant on heat stress tolerance of cucumber. Appl. Soil Ecol..

[13] Sangamesh M., Jambagi S., Vasanthakumari M., Shetty N.J., Kolte H., Ravikanth G., Nataraja K.N., Uma Shaanker R. (2018). Thermotolerance of fungal endophytes isolated from plants adapted to the Thar Desert, India. Symbiosis.

[14] Zhou W.-N., White J.F., Soares M.A., Torres M.S., Zhou Z.-P., Li H.-Y. (2015). Diversity of fungi associated with plants growing in geothermal ecosystems and evaluation of their capacities to enhance thermotolerance of host plants. J. Plant Interact..

[15] Márquez L.M., Redman R.S., Rodriguez R.J., Roossinck M.J. (2007). A virus in a fungus in a plant: Three-way symbiosis required for thermal tolerance. Science.

[16] Rodriguez R., Redman R. (2008). More than 400 million years of evolution and some plants still can’t make it on their own: Plant stress tolerance via fungal symbiosis. J. Exp. Bot..

[17] Khan M.I., Ali N., Jan G., Hamayun M., Jan F.G., Iqbal A., Hussain A., Lee I.-J. (2022). Salt stress alleviation in Triticum aestivum through primary and secondary metabolites modulation by Aspergillus terreus BTK-1. Front. Plant Sci..

[18] Bibi N., Jan G., Jan F.G., Hamayun M., Iqbal A., Hussain A., Rehman H., Tawab A., Khushdil F. (2019). *Cochliobolus* sp. acts as a biochemical modulator to alleviate salinity stress in okra plants. Plant Physiol. Biochem..

[19] Kazerooni E.A., Maharachchikumbura S.S., Al-Sadi A.M., Rashid U., Kang S.-M., Lee I.-J. (2022). Actinomucor elegans and podospora bulbillosa positively improves endurance to water deficit and Salinity Stresses in tomato plants. J. Fungi.

[20] Redman R.S., Kim Y.O., Cho S., Mercer M., Rienstra M., Manglona R., Biaggi T., Zhou X.-G., Chilvers M., Gray Z. (2021). A symbiotic approach to generating stress tolerant crops. Microorganisms.

[21] Sampangi-Ramaiah M.H., Jagadheesh Dey P., Jambagi S., Vasantha Kumari M., Oelmueller R., Nataraja K.N., Venkataramana Ravishankar K., Ravikanth G., Uma Shaanker R. (2020). An endophyte from salt-adapted Pokkali rice confers salt-tolerance to a salt-sensitive rice variety and targets a unique pattern of genes in its new host. Sci. Rep..

[22] Manasa K., Vasanthakumari M., Nataraja K., Shaanker R.U. (2020). Endophytic fungi of salt adapted Ipomea pes-caprae LR Br. Curr. Sci..

[23] Hashem A., Alqarawi A.A., Radhakrishnan R., Al-Arjani A.-B.F., Aldehaish H.A., Egamberdieva D., Abd_Allah E.F. (2018). Arbuscular mycorrhizal fungi regulate the oxidative system, hormones and ionic equilibrium to trigger salt stress tolerance in Cucumis sativus L. Saudi J. Biol. Sci..

[24] Galeano R.M.S., Silva S.M., Yonekawa M.K.A., de Alencar Guimarães N.C., Giannesi G.C., Masui D.C., Corrêa B.O., da Silva Brasil M., Zanoelo F.F. (2023). Penicillium chrysogenum strain 34-P promotes plant growth and improves initial development of maize under saline conditions. Rhizosphere.

[25] Tarroum M., Romdhane W.B., Al-Qurainy F., Ali A.A.M., Al-Doss A., Fki L., Hassairi A. (2022). A novel PGPF Penicillium olsonii isolated from the rhizosphere of Aeluropus littoralis promotes plant growth, enhances salt stress tolerance, and reduces chemical fertilizers inputs in hydroponic system. Front. Microbiol..

[26] Patel S., Parekh V., Patel K., Jha S. (2021). Plant growth-promoting activities of *Penicillium* sp. NAUSF2 ameliorate Vigna radiata salinity stress in phosphate-deficient saline soil. Appl. Biochem. Microbiol..

[27] Moghaddam M.S.H., Safaie N., Soltani J., Hagh-Doust N. (2021). Desert-adapted fungal endophytes induce salinity and drought stress resistance in model crops. Plant Physiol. Biochem..

[28] Liu Z., Xu N., Pang Q., Khan R.A.A., Xu Q., Wu C., Liu T. (2023). A Salt-Tolerant Strain of Trichoderma longibrachiatum HL167 Is Effective in Alleviating Salt Stress, Promoting Plant Growth, and Managing Fusarium Wilt Disease in Cowpea. J. Fungi.

[29] Zhang S., Gan Y., Xu B. (2016). Application of plant-growth-promoting fungi Trichoderma longibrachiatum T6 enhances tolerance of wheat to salt stress through improvement of antioxidative defense system and gene expression. Front. Plant Sci..

[30] Qiang X., Ding J., Lin W., Li Q., Xu C., Zheng Q., Li Y. (2019). Alleviation of the detrimental effect of water deficit on wheat (*Triticum aestivum* L.) growth by an indole acetic acid-producing endophytic fungus. Plant Soil.

[31] Redman R.S., Kim Y.O., Woodward C.J., Greer C., Espino L., Doty S.L., Rodriguez R.J. (2011). Increased fitness of rice plants to abiotic stress via habitat adapted symbiosis: A strategy for mitigating impacts of climate change. PLoS ONE.

[32] Bilal S., Shahzad R., Imran M., Jan R., Kim K.M., Lee I.-J. (2020). Synergistic association of endophytic fungi enhances Glycine max L. resilience to combined abiotic stresses: Heavy metals, high temperature and drought stress. Ind. Crops Prod..

[33] Sherameti I., Tripathi S., Varma A., Oelmüller R. (2008). The root-colonizing endophyte Pirifomospora indica confers drought tolerance in Arabidopsis by stimulating the expression of drought stress–related genes in leaves. Mol. Plant-Microbe Interact..

[34] Naz F., Hamayun M., Rauf M., Arif M., Afzal Khan S., Ud-Din J., Gul H., Hussain A., Iqbal A., Kim H.-Y. (2022). Molecular mechanism of Cu metal and drought stress resistance triggered by Porostereum spadiceum AGH786 in *Solanum lycopersicum* L. Front. Plant Sci..

[35] Bakhshi S., Eshghi S., Banihashemi Z. (2023). Application of candidate endophytic fungi isolated from extreme desert adapted trees to mitigate the adverse effects of drought stress on maize (*Zea mays* L.). Plant Physiol. Biochem..

[36] Zajc J., Černoša A., Sun X., Fang C., Gunde-Cimerman N., Song Z., Gostinčar C. (2022). From glaciers to refrigerators: The population genomics and biocontrol potential of the black yeast Aureobasidium subglaciale. Microbiol. Spectr..

[37] Di Francesco A., Zajc J., Gunde-Cimerman N., Aprea E., Gasperi F., Placì N., Caruso F., Baraldi E. (2020). Bioactivity of volatile organic compounds by Aureobasidium species against gray mold of tomato and table grape. World J. Microbiol. Biotechnol..

[38] Treseder K.K., Holden S.R. (2013). Fungal carbon sequestration. Science.

[39] Fellbaum C.R., Mensah J.A., Pfeffer P.E., Kiers E.T., Bücking H. (2012). The role of carbon in fungal nutrient uptake and transport: Implications for resource exchange in the arbuscular mycorrhizal symbiosis. Plant Signal Behav..

[40] Mendez-Millan M., Dignac M.-F., Rumpel C., Derenne S. (2010). Quantitative and qualitative analysis of cutin in maize and a maize-cropped soil: Comparison of CuO oxidation, transmethylation and saponification methods. Org. Geochem..

[41] Li R., Tao R., Ling N., Chu G. (2017). Chemical, organic and bio-fertilizer management practices effect on soil physicochemical property and antagonistic bacteria abundance of a cotton field: Implications for soil biological quality. Soil Tillage Res..

[42] Kuzyakov Y., Domanski G. (2000). Carbon input by plants into the soil. Review. J. Plant Nutr. Soil Sci..

[43] Ma X., Xu X., Geng Q., Luo Y., Ju C., Li Q., Zhou Y. (2023). Global arbuscular mycorrhizal fungal diversity and abundance decreases with soil available phosphorus. Glob. Ecol. Biogeogr..

[44] Bahn M., Rodeghiero M., Anderson-Dunn M., Dore S., Gimeno C., Drösler M., Williams M., Ammann C., Berninger F., Flechard C. (2008). Soil respiration in European grasslands in relation to climate and assimilate supply. Ecosystems.

[45] Friedlingstein P., Cox P., Betts R., Bopp L., von Bloh W., Brovkin V., Cadule P., Doney S., Eby M., Fung I. (2006). Climate–carbon cycle feedback analysis: Results from the C4MIP model intercomparison. J. Clim..

[46] Shi Z., Zhang L., Li X., Feng G., Tian C., Christie P. (2007). Diversity of arbuscular mycorrhizal fungi associated with desert ephemerals in plant communities of Junggar Basin, northwest China. Appl. Soil Ecol..

[47] Qi Y., Xu M., Wu J. (2002). Temperature sensitivity of soil respiration and its effects on ecosystem carbon budget: Nonlinearity begets surprises. Ecol. Model..

[48] Frąc M., Hannula S.E., Bełka M., Jędryczka M. (2018). Fungal biodiversity and their role in soil health. Front. Microbiol..

[49] Dhawi F. (2023). How can we stabilize soil using microbial communities and mitigate desertification?. Sustainability.

[50] Cameron R.E. (2013). Cold desert characteristics and problems relevant to other arid lands. Arid Lands Perspect..

[51] Apple M.E. (2010). Aspects of mycorrhizae in desert plants. Desert Plants: Biology and Biotechnology.

[52] Augé R.M. (2001). Water relations, drought and vesicular-arbuscular mycorrhizal symbiosis. Mycorrhiza.

[53] Soon N.W., Lee L.M., Khun T.C., Ling H.S. (2013). Improvements in engineering properties of soils through microbial-induced calcite precipitation. KSCE J. Civ. Eng..

[54] Mortensen B., Haber M., DeJong J., Caslake L., Nelson D. (2011). Effects of environmental factors on microbial induced calcium carbonate precipitation. J. Appl. Microbiol..

[55] Chittoori B.C., Rahman T., Burbank M. (2021). Microbial-facilitated calcium carbonate precipitation as a shallow stabilization alternative for expansive soil treatment. Geotechnics.

[56] Nemati M., Voordouw G. (2003). Modification of porous media permeability, using calcium carbonate produced enzymatically in situ. Enzym. Microb. Technol..

[57] Frąc M., Jezierska-Tys S., Yaguchi T. (2015). Occurrence, detection, and molecular and metabolic characterization of heat-resistant fungi in soils and plants and their risk to human health. Adv. Agron..

[58] Tedersoo L., Bahram M., Zobel M. (2020). How mycorrhizal associations drive plant population and community biology. Science.

[59] Al-Whaibi M.H. (2009). Desert Plants and Mycorrhizae (A mini-review). J. Pure Appl. Microbiol..

[60] Nasielski J., Furze J.R., Tan J., Bargaz A., Thevathasan N.V., Isaac M.E. (2015). Agroforestry promotes soybean yield stability and N 2-fixation under water stress. Agron. Sustain. Dev..

[61] Prasad P.V., Boote K.J., Allen L.H., Thomas J.M. (2002). Effects of elevated temperature and carbon dioxide on seed-set and yield of kidney bean (*Phaseolus vulgaris* L.). Glob. Chang. Biol..

[62] Conde A., Chaves M.M., Gerós H. (2011). Membrane transport, sensing and signaling in plant adaptation to environmental stress. Plant Cell Physiol..

[63] Lynch J.P. (2011). Root phenes for enhanced soil exploration and phosphorus acquisition: Tools for future crops. Plant Physiol..

[64] Tilman D., Balzer C., Hill J., Befort B.L. (2011). Global food demand and the sustainable intensification of agriculture. Proc. Nat. Acad. Sci. USA.

[65] United Nations Department of Economic Social Affairs. https://www.un.org/development/desa/dpad/.

[66] Van Dijk M., Morley T., Rau M.L., Saghai Y. (2021). A meta-analysis of projected global food demand and population at risk of hunger for the period 2010–2050. Nat. Food.

[67] Hochman Z., Gobbett D.L., Horan H. (2017). Climate trends account for stalled wheat yields in Australia since 1990. Glob. Chang. Biol..

[68] Del Pozo A., Brunel-Saldias N., Engler A., Ortega-Farias S., Acevedo-Opazo C., Lobos G.A., Jara-Rojas R., Molina-Montenegro M.A. (2019). Climate change impacts and adaptation strategies of agriculture in Mediterranean-climate regions (MCRs). Sustainability.

[69] Garreaud R.D., Alvarez-Garreton C., Barichivich J., Boisier J.P., Christie D., Galleguillos M., LeQuesne C., McPhee J., Zambrano-Bigiarini M. (2017). The 2010–2015 megadrought in central Chile: Impacts on regional hydroclimate and vegetation. Hydrol. Earth Syst. Sci..

[70] Williams C.J. (2017). Climate change in Chile: An analysis of state-of-the-art observations, satellite-derived estimates and climate model simulations. J. Earth Sci. Clim. Chang..

[71] Maia de Queiroz G.C., Francismar de Medeiros J., Cesar Constante D., Pires de Souza M.V., Vieira de Sousa L., de Castro Granjeiro J.C., Rafael da Silva R. (2022). Soil susceptibility to salinity and sodicity in sorghum areas under abiotic stress. Rev. Bras. Agric. Irrig..

[72] Nda M., Adnan M.S., Ahmad K.A., Usman N., Razi M.A.M., Daud Z. (2018). A review on the causes, effects and mitigation of climate changes on the environmental aspects. Int. J. Integr. Eng..

[73] Acuña-Rodríguez I.S., Ballesteros G.I., Atala C., Gundel P.E., Molina-Montenegro M.A. (2022). Hardening blueberry plants to face drought and cold events by the application of fungal endophytes. Agronomy.

[74] Garnica S., Liao Z., Hamard S., Waller F., Parepa M., Bossdorf O. (2022). Environmental stress determines the colonization and impact of an endophytic fungus on invasive knotweed. Biol. Invasions.

[75] Bertini L., Perazzolli M., Proietti S., Capaldi G., Savatin D.V., Bigini V., Longa C.M.O., Basaglia M., Favaro L., Casella S. (2022). Biodiversity and bioprospecting of fungal endophytes from the antarctic plant Colobanthus quitensis. J. Fungi.

[76] Saikkonen K., Wäli P., Helander M., Faeth S.H. (2004). Evolution of endophyte–plant symbioses. Trends Plant Sci..

[77] Tiwari P., Bae H. (2022). Endophytic fungi: Key insights, emerging prospects, and challenges in natural product drug discovery. Microorganisms.

[78] Verma S.K., Sahu P.K., Kumar K., Pal G., Gond S.K., Kharwar R.N., White J.F. (2021). Endophyte roles in nutrient acquisition, root system architecture development and oxidative stress tolerance. J. Appl. Microbiol..

[79] Mandal S.M., Chakraborty D., Dey S. (2010). Phenolic acids act as signaling molecules in plant-microbe symbioses. Plant Signal. Behav..

[80] Wang X., Li Y., Zhang X., Lai D., Zhou L. (2017). Structural diversity and biological activities of the cyclodipeptides from fungi. Molecules.

[81] Fu Y., Ma H., Chen S., Gu T., Gong J. (2018). Control of proline accumulation under drought via a novel pathway comprising the histone methylase CAU1 and the transcription factor ANAC055. J. Exp. Bot..

[82] Toghueo R.M.K., Boyom F.F. (2020). Endophytic Penicillium species and their agricultural, biotechnological, and pharmaceutical applications. 3 Biotech..

[83] Bell C.A., Magkourilou E., Barker H., Barker A., Urwin P.E., Field K.J. (2023). Arbuscular mycorrhizal fungal-induced tolerance is determined by fungal identity and pathogen density. Plants People Planet.

[84] Rosikiewicz P., Bonvin J., Sanders I.R. (2017). Cost-efficient production of in vitro Rhizophagus irregularis. Mycorrhiza.

[85] Madouh T.A., Quoreshi A.M. (2023). The function of arbuscular mycorrhizal fungi associated with drought stress resistance in native plants of arid desert ecosystems: A review. Diversity.

[86] Valentin D.N., Voyron S., Soteras F., Iriarte H.J., Giovannini A., Lumini E., Lugo M.A. (2023). Modeling geographic distribution of arbuscular mycorrhizal fungi from molecular evidence in soils of Argentinean Puna using a maximum entropy approach. PeerJ.

[87] Cantrell S.A., Dianese J., Fell J., Gunde-Cimerman N., Zalar P. (2011). Unusual fungal niches. Mycologia.

[88] Koltai H., Kapulnik Y., Seckbach G. (2010). Arbuscular Mycorrhizal Symbiosis under Stress Conditions: Benefits and Costs.

[89] Li J., Meng B., Chai H., Yang X., Song W., Li S., Lu A., Zhang T., Sun W. (2019). Arbuscular mycorrhizal fungi alleviate drought stress in C3 (Leymus chinensis) and C4 (Hemarthria altissima) grasses via altering antioxidant enzyme activities and photosynthesis. Front. Plant Sci..

[90] Díaz-Valderrama J.R., Nguyen H.D., Aime M.C. (2017). Wallemia peruviensis sp. nov., a new xerophilic fungus from an agricultural setting in South America. Extremophiles.

[91] Le Pioufle O., Declerck S. (2018). Reducing water availability impacts the development of the arbuscular mycorrhizal fungus Rhizophagus irregularis MUCL 41833 and its ability to take up and transport phosphorus under in vitro conditions. Front. Microbiol..

[92] Boerstler B., Thiery O., Sýkorová Z., Berner A., Redecker D. (2010). Diversity of mitochondrial large subunit rDNA haplotypes of Glomus intraradices in two agricultural field experiments and two semi-natural grasslands. Mol. Ecol..

[93] Picone C. (2000). Diversity and abundance of arbuscular–mycorrhizal fungus spores in tropical forest and pasture. Biotropica.

[94] Pathak H., Jain N., Bhatia A., Kumar A., Chatterjee D. (2016). Improved nitrogen management: A key to climate change adaptation and mitigation. Indian. J. Fertil..

[95] Sarkhel R., Sengupta S., Das P., Bhowal A. (2020). Comparative biodegradation study of polymer from plastic bottle waste using novel isolated bacteria and fungi from marine source. J. Polym. Res..

[96] Srikanth M., Sandeep T., Sucharitha K., Godi S. (2022). Biodegradation of plastic polymers by fungi: A brief review. Bioresour. Bioprocess..

[97] Chai Y., Bai M., Chen A., Peng L., Shao J., Luo S., Deng Y., Yan B., Peng C. (2022). Valorization of waste biomass through fungal technology: Advances, challenges, and prospects. Ind. Crops Prod..

[98] Molelekoa T.B.J., Regnier T., da Silva L.S., Augustyn W. (2021). Production of pigments by filamentous fungi cultured on agro-industrial by-products using submerged and solid-state fermentation methods. Fermentation.

[99] Huang D.-L., Zeng G.-M., Jiang X.-Y., Feng C.-L., Yu H.-Y., Huang G.-H., Liu H.-L. (2006). Bioremediation of Pb-contaminated soil by incubating with Phanerochaete chrysosporium and straw. J. Hazard. Mater..

[100] Purnomo A.S., Mori T., Kamei I., Nishii T., Kondo R. (2010). Application of mushroom waste medium from Pleurotus ostreatus for bioremediation of DDT-contaminated soil. Int. Biodeterior. Biodegrad..

[101] Rigas F., Papadopoulou K., Philippoussis A., Papadopoulou M., Chatzipavlidis J. (2009). Bioremediation of lindane contaminated soil by Pleurotus ostreatus in non sterile conditions using multilevel factorial design. Water Air Soil Pollut..

[102] Aminabhavi T., Balundgi R., Cassidy P. (1990). A review on biodegradable plastics. Polym. Plast. Technol. Eng..

[103] Sodre V., Vilela N., Tramontina R., Squina F.M. (2021). Microorganisms as bioabatement agents in biomass to bioproducts applications. Biomass Bioenergy.

[104] Pearce T.R., Antonelli A., Brearley F.Q., Couch C., Campostrini Forzza R., Gonçalves S.C., Magassouba S., Morim M.P., Mueller G.M., Nic Lughadha E. (2020). International collaboration between collections-based institutes for halting biodiversity loss and unlocking the useful properties of plants and fungi. Plants People Planet.

